# Supplementary material for: Notch signaling restricts FGF pathway activation in parapineal cells to promote their collective migration
Source: eLife. 2019 Sep 9;8:e46275. doi: 10.7554/eLife.46275 (PMC6733574; doi:10.7554/eLife.46275)
Supplement: Transparent reporting form [file elife-46275-transrepform.docx]

***eLife’s* transparent reporting form**

We encourage authors to provide detailed information *within their submission* to facilitate the interpretation and replication of experiments. Authors can upload supporting documentation to indicate the use of appropriate reporting guidelines for health-related research (see [EQUATOR Network](http://www.equator-network.org/%20)), life science research (see the [BioSharing Information Resource](https://biosharing.org/)), or the [ARRIVE guidelines](http://www.plosbiology.org/article/info:doi/10.1371/journal.pbio.1000412) for reporting work involving animal research. Where applicable, authors should refer to any relevant reporting standards documents in this form.

If you have any questions, please consult our Journal Policies and/or contact us: [editorial@elifesciences.org](mailto:editorial@elifesciences.org).

**Sample-size estimation**

- You should state whether an appropriate sample size was computed when the study was being designed
- You should state the statistical method of sample size computation and any required assumptions
- If no explicit power analysis was used, you should describe how you decided what sample (replicate) size (number) to use

Please outline where this information can be found within the submission (e.g., sections or figure legends), or explain why this information doesn’t apply to your submission:

Sample sizes were not specifically computed before starting the experiments. All sample sizes (n) are presented in Figure legends together with the p-value and statistical test used; sample sizes are also most mentioned in the results section.

**Replicates**

- You should report how often each experiment was performed
- You should include a definition of biological versus technical replication
- The data obtained should be provided and sufficient information should be provided to indicate the number of independent biological and/or technical replicates
- If you encountered any outliers, you should describe how these were handled
- Criteria for exclusion/inclusion of data should be clearly stated
- High-throughput sequence data should be uploaded before submission, with a private link for reviewers provided (these are available from both GEO and ArrayExpress)

Please outline where this information can be found within the submission (e.g., sections or figure legends), or explain why this information doesn’t apply to your submission:

- Our data are representative of 3 independent experiments when they support the most important conclusion of our study (all data with *gfi1ab* marker in Fig1, 2, 3 and epistatic experiments Fig4A-F). Data confirming an observation are usually representative of 2 independent experiments (for instance all data with *sox1a* marker that confirm data obtained with *gfi1ab*). In rare cases, data corresponds to a single experiment, usually when sample size was sufficient (for instance in Figure 2–figure supplement 1 where n=17 to 20) and/or when the data confirmed a conclusion obtained in other ways (for instance, the dot plots in Fig 3J, L present an atypical few number of embryos from one experiment (n=5 controls, n=6 NICD+ embryos); in this case, these data are supporting similar observations made at 36hpf with a nuclear staining (Fig 3K) and at 48 hpf with 3 different markers (*sox1a, gfi1ab, tbx2b*; Fig 3V-X) that all indicate significant parapineal migration defects in NICD+ embryos.
- Our independent experiments correspond to biological replicates (experiments performed on different days, on different embryo samples derived from different fish crosses). For each single replicate, embryos were collected from different set of fish crosses to increase further the heterogeneity of embryos samples within the experiment.
- Information on replicate numbers is mentioned in the corresponding figure legends.

**Statistical reporting**

- Statistical analysis methods should be described and justified
- Raw data should be presented in figures whenever informative to do so (typically when N per group is less than 10)
- For each experiment, you should identify the statistical tests used, exact values of N, definitions of center, methods of multiple test correction, and dispersion and precision measures (e.g., mean, median, SD, SEM, confidence intervals; and, for the major substantive results, a measure of effect size (e.g., Pearson's r, Cohen's d)
- Report exact p-values wherever possible alongside the summary statistics and 95% confidence intervals. These should be reported for all key questions and not only when the p-value is less than 0.05.

Please outline where this information can be found within the submission (e.g., sections or figure legends), or explain why this information doesn’t apply to your submission:

- Statistical methods are described and justified in Materials & Methods section.
- For each Figure, we report the exact p-value and statistical test used in the legend. P-value and Mean+-SD are also often included in the Result section.
- We choose to show all data as dot plots showing values for individual embryo (number or parapineal mean position) rather than as boxplot or histograms so that the reader has access to a presentation that more faithfull represents the raw data.
- When relevant (for graphs showing parapineal cell numbers for instance) we included a Mean+SEM on our dot plots. For dot plots showing parapineal mean position, we did not show Mean+SEM; in this case, the dataset are often not normal as they can be composed of a variable number of embryos with a right parapineal position in addition to the majority of embryos with left parapineal.

(For large datasets, or papers with a very large number of statistical tests, you may upload a single table file with tests, Ns, etc., with reference to sections in the manuscript.)

**Group allocation**

- Indicate how samples were allocated into experimental groups (in the case of clinical studies, please specify allocation to treatment method); if randomization was used, please also state if restricted randomization was applied
- Indicate if masking was used during group allocation, data collection and/or data analysis

Please outline where this information can be found within the submission (e.g., sections or figure legends), or explain why this information doesn’t apply to your submission:

- Samples were allocated into experimental groups relative to phenotype in the case of *midbomb* mutants (*mib*-/-) versus siblings (+/- or +/+) (Figures 1, 4A-G and Figure 2–figure supplement 1) or by PCR genotyping for allocating *mib*+/- versus *mib*+/+ (Figure 2, Figure 2–figure supplement 1, Figure 2–figure supplement 2) or NICD expressing embryos versus controls (Figure 3, Figure 2–figure supplement 2, Figure 3–figure supplement 1). Embryos carrying the *Tg(hsp70:ca-fgfr1; cryaa:DsRed)^pd3^* transgene were identified by the presence of DsRed expression in the lens from 48 hpf (Figure 4H-I). These details are found in the Materials & Methods section of our manuscript under “Fish lines”.
- To avoid bias, data in Figure 4E-F were analysed blind for DMSO versus SU5402 treatment. Data describing LY411575 treatment (Figure 2I-N, Figure 2–figure supplement 1) were analysed blind for the *mib* genotypes (*mib+/-* heterozygotes versus *+/+* controls). This is mentioned in Materials & Methods section under “Quantification”.

**Additional data files (“source data”)**

- We encourage you to upload relevant additional data files, such as numerical data that are represented as a graph in a figure, or as a summary table
- Where provided, these should be in the most useful format, and they can be uploaded as “Source data” files linked to a main figure or table
- Include model definition files including the full list of parameters used
- Include code used for data analysis (e.g., R, MatLab)
- Avoid stating that data files are “available upon request”

Please indicate the figures or tables for which source data files have been provided:

We have provided source data files for Figures 1-4.
